# Unipolar diathermy applied via the terminal pin to release an entrapped LBBAP lead helix: a case report

**DOI:** 10.1093/ehjcr/ytag477

**Published:** 2026-06-20

**Authors:** James Clark, Lewis Holmes, Owen L Williams, Lianne Easson, Victoria Brooks

**Affiliations:** Cardiology Department, Royal Glamorgan Hospital, Ynysmaerdy Terrace, Pontyclun CF72 8XR, UK; Cardiology Department, Royal Glamorgan Hospital, Ynysmaerdy Terrace, Pontyclun CF72 8XR, UK; Cardiology Department, Royal Glamorgan Hospital, Ynysmaerdy Terrace, Pontyclun CF72 8XR, UK; Cardiology Department, Royal Glamorgan Hospital, Ynysmaerdy Terrace, Pontyclun CF72 8XR, UK; Cardiology Department, Royal Glamorgan Hospital, Ynysmaerdy Terrace, Pontyclun CF72 8XR, UK

**Keywords:** LBBAP, Stylet driven lead, Helix entanglement, Novel technique, Case report

## Abstract

**Background:**

Left bundle branch area pacing (LBBAP) is increasingly being adopted as a physiological solution to both bradycardia and resynchronization indications. Deep septal positioning of both stylet-less and stylet driven leads can be complicated by unexpected entrapment in myocardial or fibrotic tissue, making advancement or removal of the lead challenging.

**Case Summary:**

A patient undergoing LBBAP implantation demonstrated suboptimal electrical parameters after initial lead advancement. Both helical retraction and counter clockwise rotation was unsuccessful in displacing the lead for a second attempt. In line with the only other case study in this situation, 3S of 40W unipolar diathermy was applied to the terminal pin, allowing electrical current to dissipate through the lead tip-tissue interface. This resulted in immediate release of the helix and successful extraction of the lead without mechanical or clinical complications.

**Discussion:**

Entrapment of the helix during LBBAP is a recognized procedural challenge but inability to overcome this with mechanical disengagement techniques is rare. This occurrence is more likely with extendable helix leads. This case demonstrates a challenging scenario whereby the helix could not be successfully retracted and the lead removed. The novel rescue technique was adapted from a previous case and reinforces the feasibility of this technique.

Learning points

1. Helical entanglement in myocardium or fibrotic tissue occurs both with stylet-less driven and stylet driven leads, and can make removal of the lead challenging.
2. Perpendicular alignment of the delivery catheter with the septal endocardium reduces the risk of lead entrapment.
3. Diathermy can be applied to the terminal pin to ablate the myocardial tissues retaining or entrapping the helix.

## Introduction

Lead entanglement leading to entrapment is a well-recognized lead behaviour in left bundle branch area pacing (LBBAP) ^1^. Lead withdrawal and subsequent repositioning is usually possible; however, emerging publications recognize the difficulty associated with damaged helixes, myocardium retained within both stylet driven and stylet-less driven helixes contributing to difficult mechanical extraction^2–4^.

An absence of knowledge exists in techniques for withdrawing an entangled lead with or without a damaged helix beyond mechanical methods. Currently, a single case report describes the use of unipolar diathermy to the terminal pin utilized in extracting an acutely retained lead .^5^

We report a case in which a brief application of unipolar diathermy delivered to the terminal pin allowed safe release of an entrapped helix on a stylet driven lead after mechanical methods failed.

## Summary figure

A suggested process to reduce the occurrence of entanglement and to increase the chance of succesful lead withdrawel based on a summary of evidence from this case report and the following references^2–6^

**Figure:**
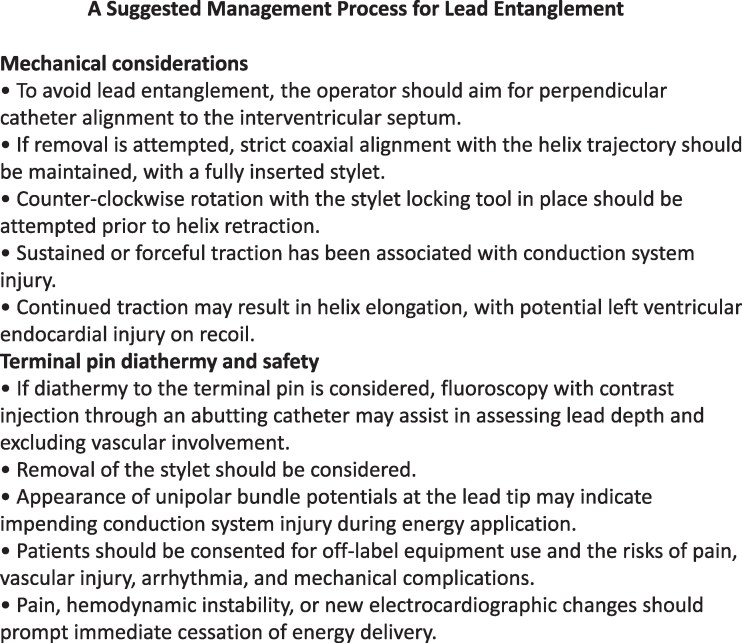


## Case report

The patient is an 87-year-old male, who had a pacing indication for exertional intolerance with atrial fibrillation and atrioventricular block on ambulatory monitoring. His resting electrocardiogram demonstrated atrial fibrillation with right bundle branch block (RBBB) (144 ms).

He attended for a day case procedure. The patient was prepared and draped with anaesthetic administered to the left infraclavicular region. Axillary access was obtained in a conventional manner and a 10.5Fr sheath placed.

**Figure 1:**
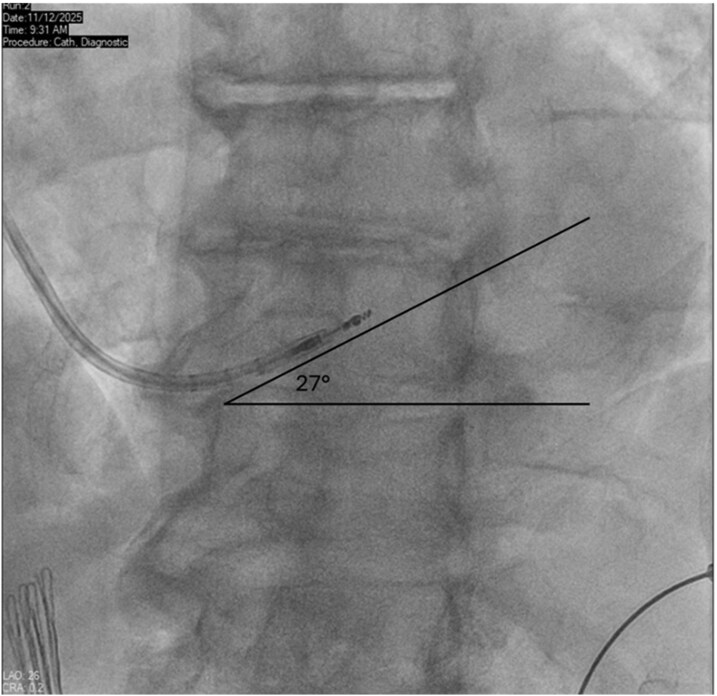
The initial LAO angulation of the catheter and lead in an LAO projection. The angle is labelled 27° to the horizontal line, with steeper angles being associated with a higher risk of lead retention on attempted retraction.

An Abbott CPS Direct 3D delivery catheter was advanced to the right ventricle. The catheter was angulated at around 30 degrees to the horizontal in the left anterior oblique view as seen in *Figure 1*. An Abbott Ultipace 69 cm lead was advanced and the helix deployed with the helix locking tool attached. After four lead rotations, and after a drop of around 50% of current of injury amplitude, and an impedance of 484 ohms, the LV-activation time was measured at 84 ms and the pacing capture threshold (PCT) was >2 V. The decision was made to reposition the lead, performed by withdrawal of the helix under fluoroscopic guidance and then counterclockwise lead rotation (CCWLR). Sequential CCWLR, advancement, and withdrawal of the lead helix was met with failure despite catheter abutment and perpendicular myocardial alignment.

**Figure 2:**
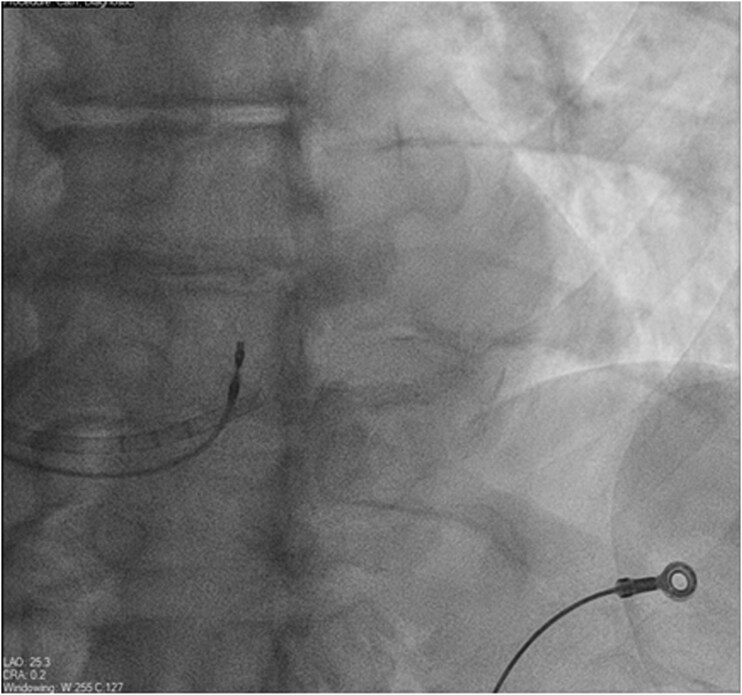
The retained lead is seen on the left of the screen, hanging freely from the RV endocardium in the LAO projection. This can be more easily appreciated in the supplementary video. 1 cm inferiorly the delivery catheter is abutting the endocardium at an appreciably more perpendicular angle than observed in *Figure 1*.

A literature search was conducted by an attendant company representative whilst a second lead was placed in a more antero-inferior position and the first lead was temporarily abandoned. The initial catheter placement for the second lead, and the final lead position can be viewed in *Figure 2*. A supplementary video is available demonstrating fulcrum sign on the functioning lead, and the retained lead moving in synchronization with the helix, indicating its relatively RV endocardial position after attempted retraction of the helix.

A single case study was identified, describing diathermy of the distal pin of the pacing lead.^5^ An injection of 3 ml contrast was administered to ensure absence of vascular perforation. No right bundle potential was visualized as expected in RBBB and fluoroscopy indicating a relatively superficial position.

It was explained to the patient that the first lead is not adequately functioning. If the lead was to be retained, there is a risk of late displacement and the helix may cause myocardial injury, there is a small increased risk of infection, there is a risk of lead–lead interaction and he would not be able to undergo magnetic resonance imaging in the future.

The second option is to diathermy the distal pin, with the intention of freeing the lead. There is a risk of pain, injury to vascular structures, including myocardial infarction, arrhythmia, and injury to the conducting system or damage to the pacing lead.

The patient gave verbal consent for the diathermy approach. A 3 s application of 40 W unipolar diathermy on ‘cut’ was administered with gentle retraction on the lead. Within the 3 s application of diathermy the lead displaced.

**Figure 3:**
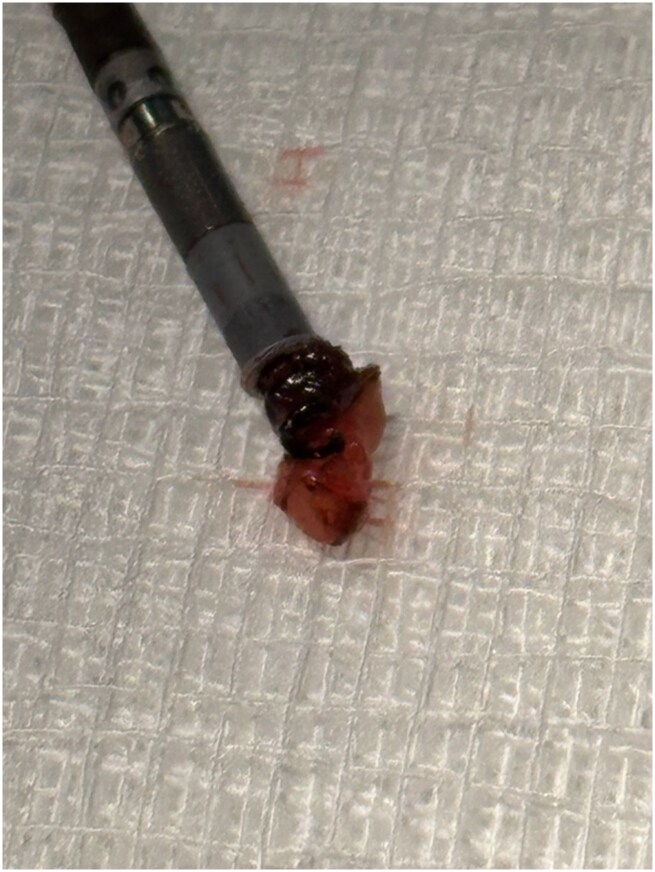
The intact lead is shown with the helix enveloped in myocardial tissue. The tissue immediately abutting and entrapped within the helix is charred. The myocardial tissue can be seen to be encroaching into the lead housing.

On examination, the helix appeared to be restricted by myocardium being withdrawn into the lead housing. The helically opposed myocardium was charred. This can be viewed in *Figure 3*.

**Figure 4:**
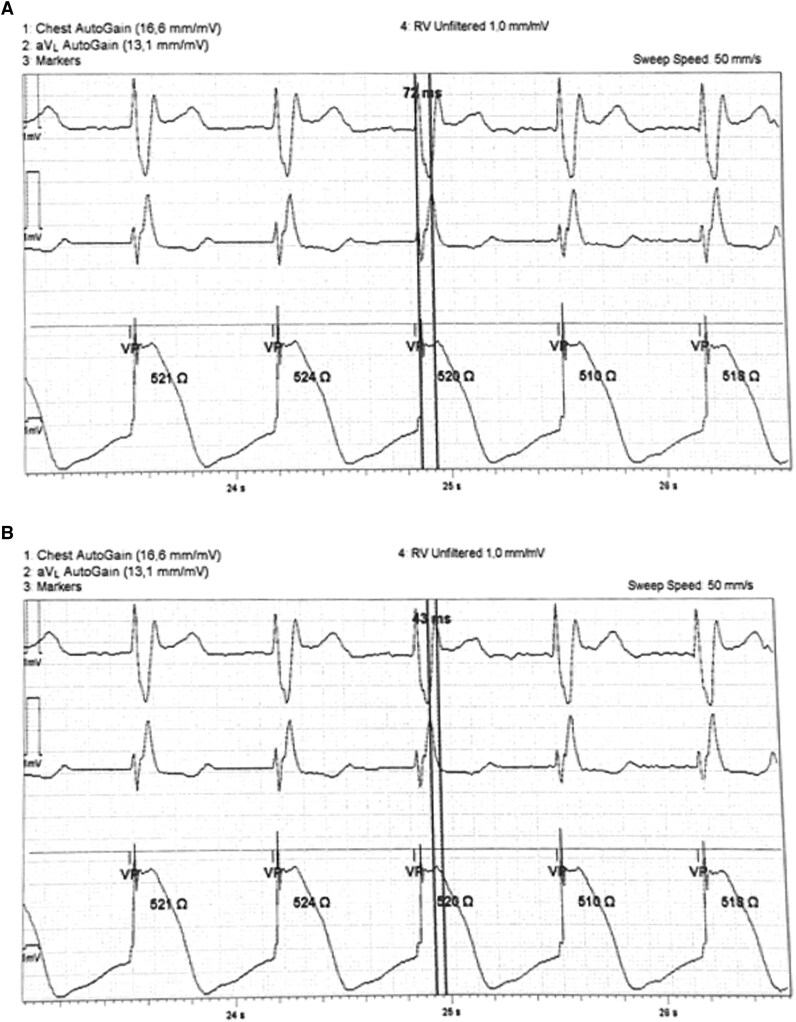

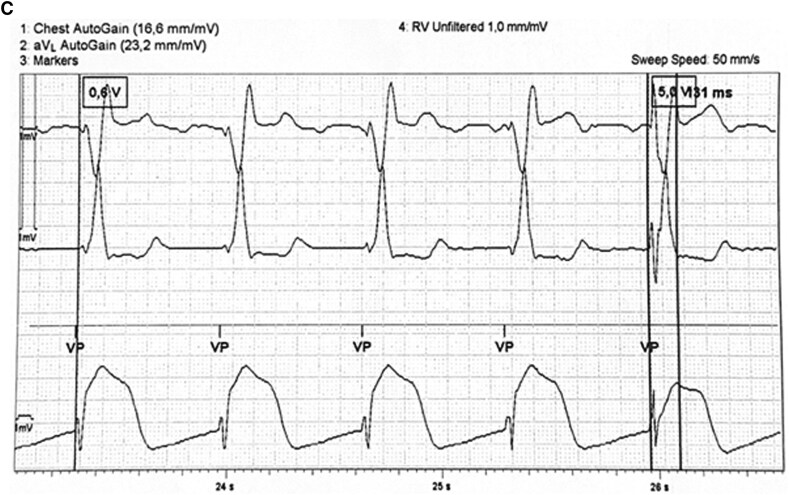
Intracardiac EGMS demonstrating left bundle branch capture and the change from non-selective to selective capture between low and high outputs. 1) Chest = V1. 2) aVl = V6. 3) markers. 4) unipolar unfiltered signal 50 mm/s. (*A*) Unipolar pacing at 5.0 V and pulse width 0.4 ms with LV activation time marked as 72 ms. (*B*) Unipolar pacing at 5.0 V and pulse width 0.4 ms with V1-V6 interpeak time marked as 43 ms. (*C*) A morphology change with inclusion of V6 s-wave at 0.6v@ 0.4 ms (left of screen), and a different morphology with loss of V6 s-wave at 5.0 V @0.4 ms.

The functioning lead was again tested in unipolar, with a lead impedance of 520 ohms, a 72 ms, an interpeak of >40 ms and evidence of a morphology change and a drop in R wave peak time in V1 when switching between 0.6 V and 5.0 V thresholds, indicating selective LBBAP as seen in *Figure 4A–C*. The lead was also tested in bipolar with a satisfactory result (R wave 7.6 mV, impedance 670 ohms, PCT of 0.6 mV).

After implantation of the device, the incision was closed. There were no immediate complications, and bedside transthoracic echocardiography did not identify any pericardial effusion. The patient was discharged later that day. Six-week follow-up yielded stable parameters, with an R wave >12 V, impedance of 510 ohms, and a PCT of 0.75 mV with 66% ventricular pacing and evidence of non-selective LBBAP capture and an improvement in his exertional ability.

## Discussion

As LBBAP implant volumes increase, so too does recognition of procedure-specific complications, particularly those related to lead behaviour within the interventricular septum.^7^ Lead entanglement, helix deformation or angulation, and failure of helix retraction are increasingly reported and this appears to be more readily seen in stylet-driven leads, with a >20-fold increased odds compared with lumen-less leads.^2,4^ Evidence from porcine heart models shows a 7-fold increase in helix deformation with non-coaxial septal alignment, as in our case, and a twofold higher risk when helical retraction is attempted before lead body rotation, particularly leading to helix extension.^1,3,4^ Myocardium retained within the helix or lead lumen can prevent full helix retraction and renders further counter-rotation ineffective.^2^ The lead may become irretrievable, potentially necessitating abandonment or referral for complex mechanical extraction, which is disproportionate in the setting of a freshly implanted pacing lead.^4^ Whilst high success rates are reported, the risk of retained fragments remains.^8^

A single prior case report described successful use of unipolar diathermy applied to the distal terminal pin to release an entrapped LBBAP lead, postulating a local ablative effect at the helix–myocardial interface.^5^

In the present case, a brief (3-s), low-power (40 W) application of unipolar diathermy in ‘cut’ mode resulted in immediate release of the lead, with no haemodynamic instability, arrhythmia, or evidence of myocardial or vascular injury. Examination of the extracted lead provided corroborative evidence: The presence of charred myocardium adherent to the non-retractable helix strongly supports the hypothesis that diathermy facilitated release by ablating entrapped tissue. This mechanism is conceptually similar to radiofrequency-assisted lead extraction techniques used in chronic transvenous lead removal, or in difficult transeptal access.^9,10^

Potential complications may include pain, myocardial injury, coronary or septal vessel damage, conduction system injury, and interaction with adjacent leads, thus terminal pin diathermy should be regarded strictly as a bailout strategy. An evidence-based approach detailing considerations and a work process for retained leads can be viewed as the summary figure. The lead has been returned to the manufacturing company for evaluation.

## Conclusion

This case adds to the limited but growing body of evidence suggesting that terminal pin diathermy may represent a viable, controlled, and effective option for releasing acutely entrapped LBBAP leads with damaged or non-retractable helices, potentially avoiding lead abandonment or more invasive extraction procedures. Further collective experience will be required before this technique can be formally incorporated into procedural algorithms.

## Supplementary Material

ytag477_Supplementary_Data

## Data Availability

The data underlying this article will be shared on reasonable request to the corresponding author.

## Lead author biography

Dr Clark is a Consultant Cardiologist working at Royal Glamorgan Hospital in Wales with an interest in left bundle branch area pacing.

## Notes

**Consent:** Written informed consent has been obtained and recorded on the EHJCR consent form and has been retained in the hospital of authorship.
